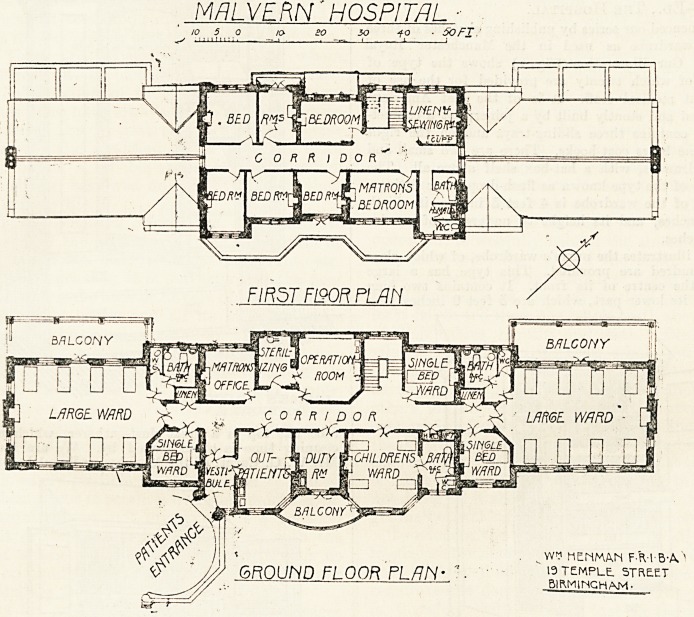# Hospital Architecture and Construction

**Published:** 1911-10-21

**Authors:** 


### HOSPITAL ARCHITECTURE AND CONSTRUCTION.

[Communications on this subject should be marked "Architecture" in the left-hand top corner of the envelope.]

### Malvern Hospital.

This new hospital, which was opened in June last, is built on the slope of a hill overlooking the Manor Park and having magnificent views of the surrounding country. Although the actual site of the hospital is not large, the immediate surroundings are and must always remain open; the conditions, therefore, as to air and prospect are as favourable as can be imagined.

The building is, as will be seen from the plan, one straight block. By taking advantage of the slope in the ground there are practically two ground floors, the lower one containing the kitchen, offices, and stores and the dining-rooms and sitting-rooms for staff; and the upper one containing the wards, with their offices. The wards are five in number, namely, two wards for eight beds each, a children's ward for four cots, and three single-bed wards. In the centre on the north-west side is the operation-room, with sterilising-room communicating; adjoining the latter is the matron's office, and on the other side of the operation-room is the staircase and a small ward. On the opposite side of the corridor are placed the patients' entrance, out-patients' room, duty-room, children's ward, and bath-room.

A serious blot on an otherwise excellent plan is the absence of any attempt to disconnect the sanitary offices from the wards. It is true that a lobby is interposed between the ward and the w.c., but it is neither lighted nor ventilated. Moreover there is no sink-room shown on the plan, and, apparently, no provision for emptying and cleansing bed-pans; unless, indeed, the circular fittings shown in the bath-rooms indicate bed-pan sinks. We can hardly conceive the possibility of any architect in these days regarding the bath-room as a suitable place for emptying bed pans. It would appear from the plan that by a relatively simple reconstruction the baths and lavatories might be placed in sanitary towers, and the present space allotted to them used as lobbies of communication.

On an upper floor over the central portion are six bedrooms for nurses and servants, the matron's bedroom, linen-room, bath-room, housemaid's closet, and w.c. In the lower ground floor are the kitchen offices, nurses' sitting-room and dining-room, wash-house, drying room, and an *x*-ray room. The architect for the hospital is Mr. William Henman, F.R.I.B.A., of Birmingham.

## Figures and Tables

**Figure f1:**